# Respiratory and systemic monocytes, dendritic cells, and myeloid‐derived suppressor cells in COVID‐19: Implications for disease severity

**DOI:** 10.1111/joim.13559

**Published:** 2022-08-31

**Authors:** Sara Falck‐Jones, Björn Österberg, Anna Smed‐Sörensen

**Affiliations:** ^1^ Division of Immunology and Allergy Department of Medicine Solna Karolinska Institutet Karolinska University Hospital Stockholm Sweden

**Keywords:** airways, COVID‐19, dendritic cells, monocytes, myeloid‐derived suppressor cells

## Abstract

Since the beginning of the SARS‐CoV‐2 pandemic in 2020, researchers worldwide have made efforts to understand the mechanisms behind the varying range of COVID‐19 disease severity. Since the respiratory tract is the site of infection, and immune cells differ depending on their anatomical location, studying blood is not sufficient to understand the full immunopathogenesis in patients with COVID‐19. It is becoming increasingly clear that monocytes, dendritic cells (DCs), and monocytic myeloid‐derived suppressor cells (M‐MDSCs) are involved in the immunopathology of COVID‐19 and may play important roles in determining disease severity. Patients with mild COVID‐19 display an early antiviral (interferon) response in the nasopharynx, expansion of activated intermediate monocytes, and low levels of M‐MDSCs in blood. In contrast, patients with severe COVID‐19 seem to lack an early efficient induction of interferons, and skew towards a more suppressive response in blood. This is characterized by downregulation of activation markers and decreased functional capacity of blood monocytes and DCs, reduced circulating DCs, and increased levels of HLA‐DR^lo^CD14^+^ M‐MDSCs. These suppressive characteristics could potentially contribute to delayed T‐cell responses in severe COVID‐19 cases. In contrast, airways of patients with severe COVID‐19 display hyperinflammation with elevated levels of inflammatory monocytes and monocyte‐derived macrophages, and reduced levels of tissue‐resident alveolar macrophages. These monocyte‐derived cells contribute to excess inflammation by producing cytokines and chemokines. Here, we review the current knowledge on the role of monocytes, DCs, and M‐MDSCs in COVID‐19 and how alterations and the anatomical distribution of these cell populations may relate to disease severity.

## Graphical abstract

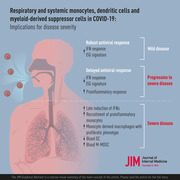

## Introduction

Infection with severe acute respiratory syndrome coronavirus 2 (SARS‐CoV‐2), the cause of the coronavirus disease of 2019 (COVID‐19), leads to respiratory illness of varying severity. Immunopathology clearly plays an important role in COVID‐19, with low lymphocyte counts, increased neutrophil‐to‐lymphocyte ratio, and exhaustion and reduction of T cells being hallmarks of severe disease [1, 2, 3]. Moreover, the dynamics of innate immune cell composition, T‐cell frequencies, and antibody responses are impacted by the progression of disease development, making sampling time a critical variable when studying pathogenesis [4, 5, 6].

Monocytes and dendritic cells (DCs) are innate immune cells that circulate in blood and line the epithelial surface of the respiratory tract where they are important sensors and responders to potential threats [7, 8, 9]. Recruitment of monocytes and DCs to the respiratory tract has been demonstrated in several human respiratory viral infections including influenza virus, respiratory syncytial virus (RSV), and hantavirus infection [10, 11, 12]. Less is known about monocytic myeloid‐derived suppressor cells (M‐MDSCs)—another innate immune cell subset—in acute respiratory viral infections, as these cells have mostly been studied in chronic inflammatory conditions such as cancer and HIV, and vaccination [13, 14]. While DCs and monocytes respond rapidly to viruses by secreting proinflammatory cytokines and inducing virus‐specific T‐cell responses [8, 15, 16, 17, 18, 19], M‐MDSCs instead suppress T‐cell proliferation [20]. Studies suggest that monocytes, DCs, and M‐MDSCs are involved in COVID‐19 pathogenesis, and that the phenotype and function of these cells differ depending on disease severity but also anatomical location [21, 22, 23, 24, 25, 26, 27, 28].

A limitation when comparing data from different studies is the lack of a uniform definition of COVID‐19 severity and at which timepoint severity is assessed [29]. Most COVID‐19 scoring systems used—including the scores developed by the WHO—are based on the degree of respiratory failure [22, 30, 31, 32], in some cases taking into account subsequent multi‐organ failure [30], or radiologic findings [32, 33]. Furthermore, the partial pressure of oxygen/fraction of inspired oxygen index has been shown to predict mortality in patients with COVID‐19 [34]. Despite varying criteria for intensive care unit admission, this is also frequently used as a marker of severe disease [27, 35]. These potential confounders should be taken into account when comparing data across studies investigating monocytes, DCs, and M‐MDSCs in patients with COVID‐19. Nevertheless, summarizing studies investigating the role of myeloid cell subsets in blood and airways during COVID‐19 in relation to disease severity is important to improving our understanding of subsequent virus‐specific adaptive immune responses needed to clear infection and provide long‐term memory.

## Sampling the human respiratory tract

**Fig. 1:**
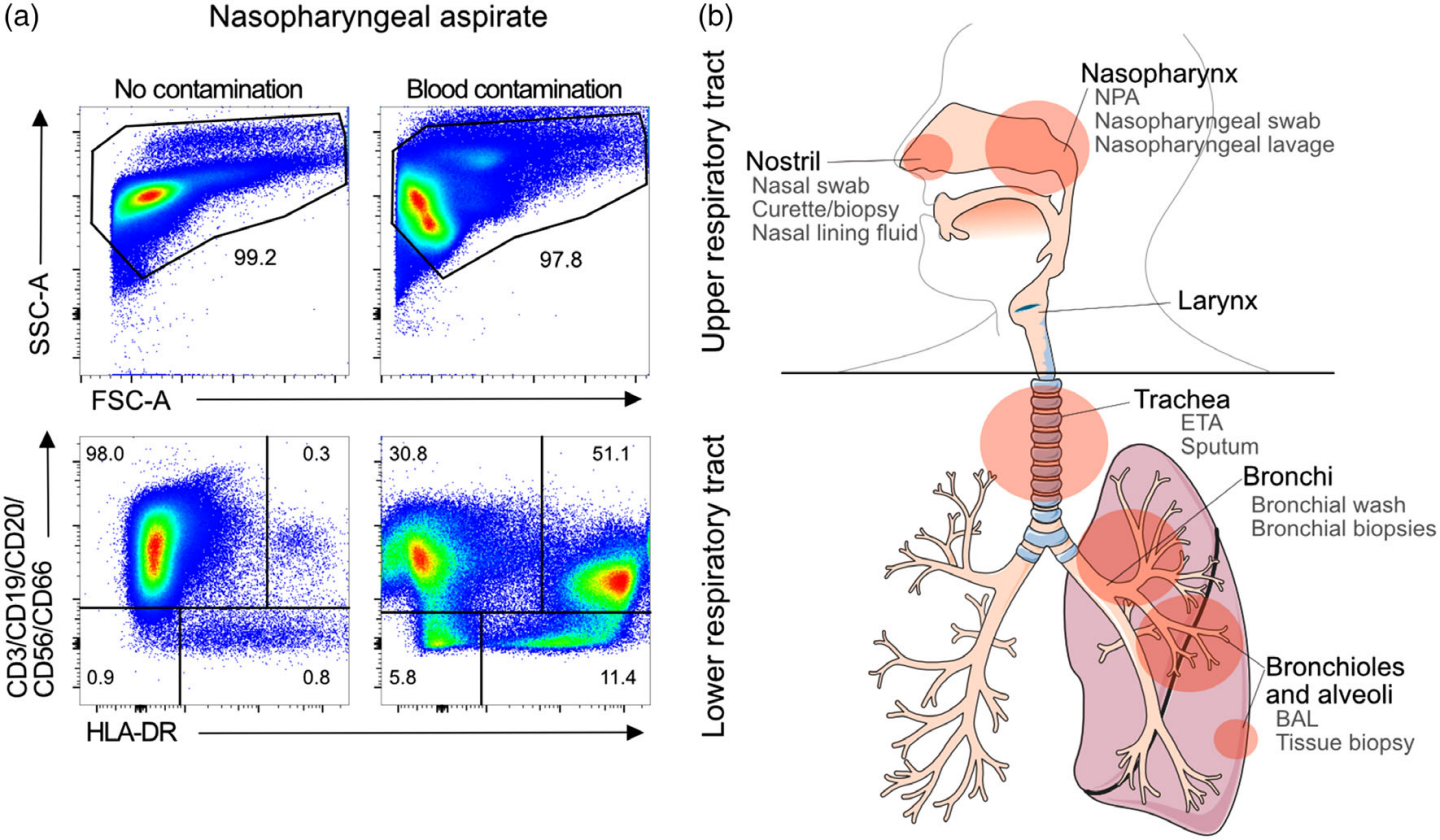
Blood contamination of nasopharyngeal aspirate and overview of different compartments and sample types in the human respiratory tract. (a) Representative flow cytometry plots of nasopharyngeal aspirate cells. Levels of CD3/CD19/CD20/CD56/CD66 positive cells in samples with and without blood contamination. (b) Overview of the human respiratory tract including the upper respiratory tract (nostril, nasopharynx, and larynx) and the lower respiratory tract (trachea, bronchi, bronchioles, and alveoli). BAL, bronchoalveolar lavage; ETA, endotracheal aspirate; NPA, nasopharyngeal aspirate.

Respiratory sampling in COVID‐19 patients is challenging compared to blood sampling for several reasons including technical difficulties, discomfort, and risk of viral transmission [36]. Furthermore, local bleeding can occur, affecting sample quality with increased proportions of blood leukocytes hiding the true mucosal immune cell composition (Fig. 1a) [12].

The upper respiratory tract comprises the area from the nostrils and the mouth to the supraglottic larynx (Fig. 1b). Nasal wash (NW) or nasopharyngeal aspirates (NPA) are suitable methods to collect live, luminal cell populations [10, 26, 37], while swabbing the nostril or nasopharynx is commonly used for soluble mediators or RNA analyses [6]. The lower respiratory tract includes the trachea, bronchi, bronchioles, and the alveoli (Fig. 1b). Endotracheal aspirates (ETA) are typically performed in intubated patients by inserting a suction catheter into the endotracheal tube. Sputum samples can also be obtained from the lower airways, by coughing up material into a sterile container. In healthy individuals, or in individuals with diseases lacking a productive cough, sputum can be induced by inhaling a saline mist. Kocbach Bølling et al. demonstrated that live immune cells (primarily macrophages) can be obtained from healthy individuals by induced sputum [38]. During a bronchoscopy, bronchial wash (BW) and bronchoalveolar lavage (BAL) samples can be collected alongside tissue biopsies [39, 40]. Lung tissue samples are typically only collected post‐mortem or after lung resection [41].

Several limitations inherent to studying immune cells from these compartments exist, including finding appropriate control groups, and functional experiments with airway immune cells from both NPA and ETA are challenging since they can be difficult to freeze viably or obtain in sufficient numbers [12]. Despite the challenges of obtaining airway samples, some studies have investigated local immune responses in the airways during COVID‐19 [26, 28, 32, 33, 42].

## Distribution, phenotype, and function of monocytes, DCs, and M‐MDSCs

Monocytes are innate immune cells of myeloid origin that are found in circulation but also in tissues, either maintaining a monocyte phenotype or differentiating into macrophages or DCs [18, 43]. In human blood, three linearly differentiated monocyte subsets, with overlapping but distinct functions, are typically defined based on CD14 and CD16 expression: CD14+CD16− classical monocytes (CM), CD14+CD16+ intermediate monocytes (IMs), and CD14−CD16+ nonclassical monocytes (NCM) [8, 19]. CMs, the most abundant subset in blood, express high levels of the migratory receptor CCR2 enabling egress from the bone marrow and quick migration to sites of inflammation/infection [7, 8, 19]. A small fraction of CMs differentiate into IMs, which have increased expression of MHC class II [8, 19] and expand during inflammatory conditions including dengue virus infection and sepsis [44, 45, 46, 47]. In contrast, NCMs remain in the vasculature and are believed to be the most differentiated subset with primarily patrolling functions [19, 44, 48, 49].

Lung macrophages include the highly abundant alveolar macrophages and the less‐studied interstitial macrophages found in lung parenchymal tissue [41, 50]. Although lung macrophages are self‐renewing distinct populations, CMs can also differentiate into alveolar and interstitial macrophages and NCMs into macrophages in the lung vasculature [49].

DCs can be found in both blood and tissue, including the respiratory tract [40], and have the unique capacity to activate naïve T cells, thus bridging innate and adaptive immunity [15, 16, 17]. DCs can be divided into myeloid conventional DCs (cDCs) and plasmacytoid DCs (pDCs). cDCs are potent antigen‐presenting cells and can be subdivided into CD141^+^ cDC1 and CD1c^+^ cDC2 [51]. cDC2 are a heterogenous group of cells that may be further subdivided into CD5^+^CD163^–^ DC2 and CD5^–^CD163^+^ DC3 [52, 53]. pDCs are potent producers of type I interferons (IFN) due to their high and constitutive expression of toll‐like receptors 7 and 9 (sensing single‐stranded RNA and double‐stranded DNA, respectively) and their downstream signaling mediators [54, 55]. Relatively recent data support that pDCs develop from a lymphoid, and not a myeloid precursor, and that cDC potential—including antigen‐presenting capacity in pDC cultures—may be a result of pre‐cDC contamination rather than a feature of pDCs themselves [56, 57].

During inflammatory conditions, MDSCs—displaying an immature phenotype and T‐cell suppressive capacity—can increase in blood [13, 14, 20]. Two main subpopulations of MDSCs with partly overlapping functions have been identified: monocytic MDSCs (M‐MDSCs) and polymorphonuclear MDSCs [58]. MDSCs suppress T cells by several mechanisms, including depletion of L‐arginine through secretion of arginase 1 (Arg‐1) and iNOS, generation of ROS, direct engagement of T‐cell inhibitory and apoptotic receptors, and production of inhibitory cytokines such as IL‐10 and TGF‐β [13, 14, 20, 59].

It is important to acknowledge that the steady‐state composition and function of immune cells differ between blood and airways, but also between different parts of the airways [40, 41, 60, 61, 62]. NW samples mainly consist of granulocytes [37], whereas the most abundant immune cell type in BW and BAL samples is alveolar macrophages [40]. The composition of monocyte subsets in BAL differs from blood, with IMs being more frequent in the lungs [63].

## Altered frequencies and activation status of peripheral monocytes and DCs during acute COVID‐19

The importance of monocytes, DCs, and M‐MDSCs in COVID‐19 was suggested early in the pandemic and has been supported by several studies [26, 27, 32, 64]. CM frequencies are reduced [24, 28, 65] or unchanged [27], whereas the IMs increase [22, 25, 27, 28, 66], but this may be less pronounced in patients with severe COVID‐19 [22, 27, 42]. Levels of NCMs decrease in a severity‐dependent manner [25, 28, 42, 67]. However, considering kinetics is important. Using mass cytometry, Chevrier et al. found a cluster of activated IMs that expanded early during disease in patients with mild COVID‐19. In patients with severe disease, two other clusters of NCMs and IMs instead increased later in the disease course [4].

Frequencies and absolute numbers of blood pDCs and DCs are reduced in COVID‐19 patients [24, 27, 28, 42, 68], especially cDC1 and pDCs [68]. This may be associated with disease severity, and pDCs were significantly more reduced in hospitalized COVID‐19 patients compared to asymptomatic individuals [69]. Frequencies of the newly identified DC3 subset were reported to be initially reduced but recovered over time, in association with seroconversion [27]. Winheim et al. found a shift towards a more inflammatory phenotype (CD14+) within the DC3 population in COVID‐19 patients [68].

**Fig. 2:**
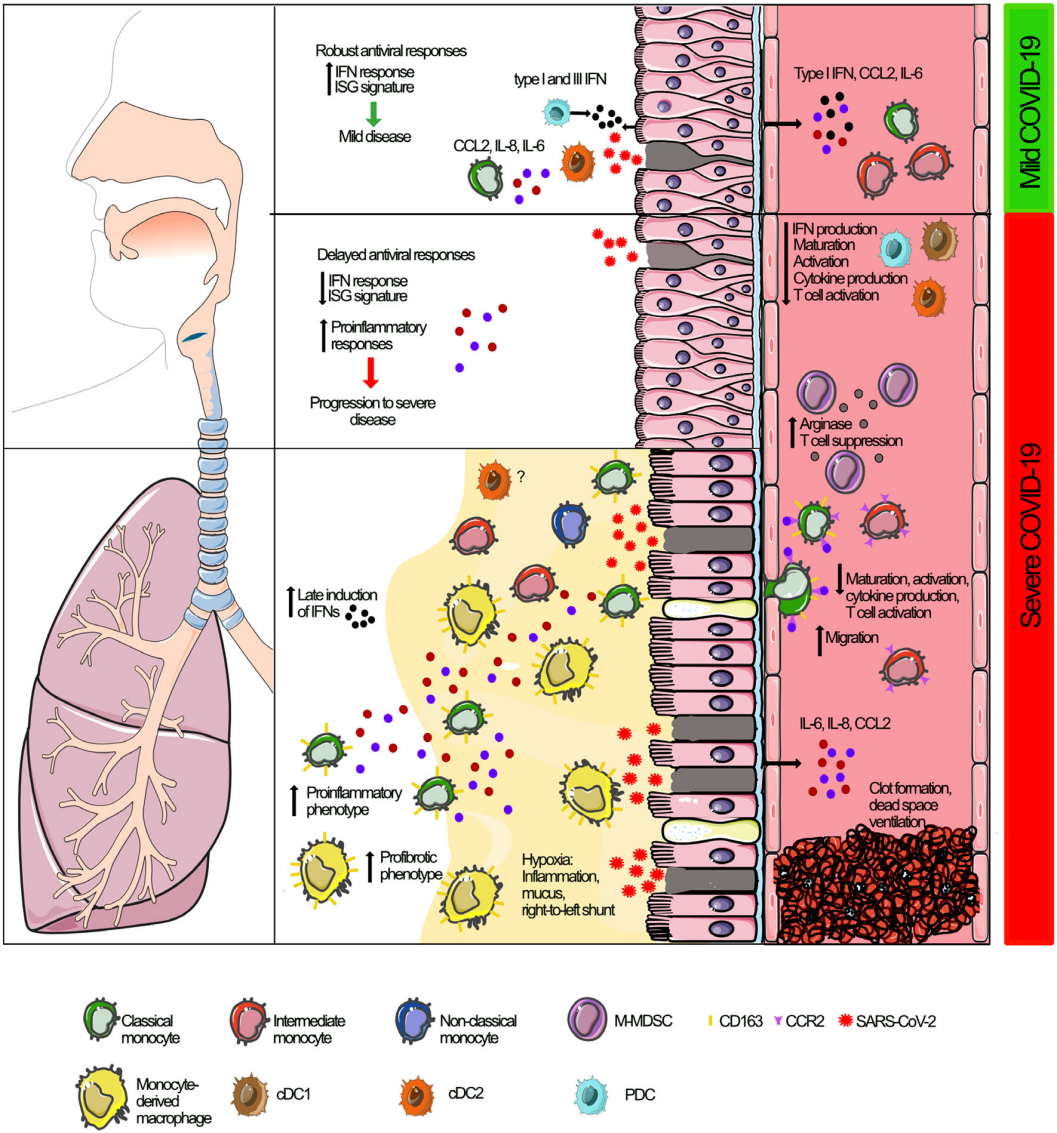
Monocytes, DCs, and M‐MDSCs in airways and blood during mild and severe COVID‐19. Overview of the different anatomical compartments, upper and lower airways (left), and blood (right) depending on COVID‐19 severity (mild or severe). cDC, conventional dendritic cell; IFN, interferon; ISG, IFN‐stimulated gene; M‐MDSC, monocytic myeloid‐derived suppressor cell; pDC, plasmacytoid dendritic cell.

In patients with COVID‐19, a severity‐dependent decrease is found in activation and maturation markers including HLA‐DR and CD86 as well as an increase in the expression of the regulatory molecule PD‐L1 on both monocytes and DCs, especially in severe COVID‐19 [4, 21, 27, 65, 68]. Some of these changes could be replicated when cDC2s were exposed to SARS‐CoV‐2 in vitro, suggesting that at least some of these changes were a direct effect of the virus [70]. Furthermore, studies show upregulation of the hemoglobin scavenger receptor CD163 on circulating monocytes and DC3s in patients with severe COVID‐19, possibly reflecting increased maturation [27, 32, 68]. Together, these studies suggest significant alterations in the frequencies of monocytes and DCs in blood, with a marked reduction of DCs (Fig. 2 and Table 1).

**Table 1:** Changes in monocyte, dendritic cell, and MDSC frequency and function in COVID‐19 patients compared to healthy controls. Green = mild disease, red = severe disease, black = no distinction between mild and severe

		Blood	Lower airways	Upper airways
CM	Freq.	− [27] − ↓ [28]	↑ [32]	− [12]
	Function	↓ Cytokine production, ↓ HLA‐DR [21], ↓ HLA‐DR [25, 27], ↑CCR2 [27], ↓ CD86 [27, 68], ↓ ability to stimulate naive T cells [68], ↑CD163 [32]	Profibrotic phenotype [97], monocyte‐derived macrophages: ↓CD163 (rel. alveolar macrophages) [32]	ND
IM	Freq.	↑↑ ↑ [27], ↑↑ − [25, 28], ↓ ↑(initially↓) [4]	↑ Rel. blood [28], ↓ [32]	− [12]
	Function	↓ HLA‐DR, ↓ CD86, ↑CCR2 [27]	↑CD40 (rel. blood) [28]	ND
NCM	Freq.	↓ ↓↓ [27, 28], ↓ ↑(initially↓)[4], − ↓ [25]	↑ Rel. blood [28], ↓ [32]	− [12]
	Function	↑ HLA‐DR [27]	↑↑CD40 (rel. blood) [28]	ND
cDC2	Freq.	↓ [27, 74] ↓ ↓ [28]	↑ (Rel. blood) [28]	↑ [12]
	Function	↓ CD86 [27, 68] ↓ HLA‐DR [27] ↓maturation markers, cytokine production and T‐cell proliferation [24], ↓IFNα and TNFα production [21]	↑CD40 (rel. blood) [28]	ND
DC3	Freq.	↓ [27, 68]	ND	ND
	Function	↓ HLA‐DR [27], ↓ CD86, ↑CCR2, ↑ inflammatory phenotype (CD14+), ↓ ability to stimulate naive T cells [68]	ND	ND
cDC1	Freq.	↓ [27], ↓ ↓ [28]	– (rel. Blood) [28]	− [12]
	Function	↓ CD86, ↓maturation markers, cytokine production and T‐cell proliferation [24], ↓IFN‐α and TNF‐α production [21]	ND	ND
pDC	Freq.	↓ [27], ↓↓ ↓↓ [28, 74]	↑ − [33]	↑ [12]
	Function	↓ CCR2 [68], ↓IFN‐α and TNF‐α production, ↓MTOR signalling [21], ↓ ↓↓ IFN‐α production [74]	↑IFN‐signalling, ↓ HLA‐DQA2 transcript levels [27]	↓ ↓↓IFN production [12, 95]
M‐MDSC	Freq.	↑ ↑↑ [24, 26, 27]	↑ [32], ↓ Rel. blood [26]	− − [26]
	Function	T‐cell suppression [26]	ND	ND

Abbreviations: cDC, conventional dendritic cell; CM, classical monocyte; DC, dendritic cell; Freq., frequency; IFN, interferon; IM, intermediate monocyte; M‐MDSC, monocytic myeloid‐derived suppressor cell; NCM, nonclassical monocyte; ND, no data; pDC, plasmacytoid dendritic cell; Rel., relative.

Comparing COVID‐19 to influenza to address whether certain immune profiles are unique to SARS‐CoV‐2 may be relevant due to the similar clinical presentation and route of transmission [71]. Mudd et al. found lower numbers of blood monocyte subsets during COVID‐19 compared to severity‐matched influenza patients [65]. Similarly, we found that IMs in both blood and the nasopharynx increased more in influenza patients than in COVID‐19 patients, both with relatively mild disease [12]. Mudd et al. also found lower cytokine levels in COVID‐19 patients, contrary to the narrative that COVID‐19 leads to a cytokine storm [65].

Interestingly, during sepsis, the pattern is more similar to that observed in COVID‐19, with decreased levels of circulating cDCs and pDCs; reduced expression of HLA‐DR, CD80, and CD86; and decreased antigen‐presenting capacity. Monocytes also have reduced expression of HLA‐DR, and M‐MDSCs are increased [72]. Altered function of cDCs and increased levels of MDSCs have been suggested to contribute to the pathogenesis in sepsis [72].

## Function of peripheral monocytes and DCs is impaired during acute COVID‐19

The function of blood monocytes and cDCs is affected during acute COVID‐19, with decreased cytokine production in response to stimulation [21, 64], especially during severe disease [64]. Mann et al. found increased TNF production upon lipopolysaccharide stimulation in CD14+ monocytes from patients with mild disease, but not in patients with more severe disease, and reduced IL‐1β production in patients compared to controls [22]. The authors suggested an altered monocyte state due to emergency myelopoiesis, supported by increased expression of the cell‐cycle marker Ki‐67 [22]. Furthermore, mixed lymphocyte reaction assays indicated that cDCs from patients with COVID‐19 have impaired T‐cell‐activating capacity [24].

When stimulated, cDCs isolated both during acute illness and early convalescence upregulated the costimulatory molecules CD80 and CD86 and produced IFN‐α and IFN‐β significantly less than cDCs from healthy controls [24]. Winheim et al. demonstrated that DC3s and CMs isolated from patients with COVID‐19 had reduced capacity to costimulate autologous naïve CD4 T cells compared to controls [68]. Stimulation with anti‐CD28 antibody generated similar proliferation in T cells from patients and controls, indicating that it was indeed a lack of costimulatory capacity from APCs that resulted in different T‐cell responses [68].

In SARS‐CoV‐2 infection, type I IFN production from pDCs seems to be impaired [73, 74], possibly due to impaired mTOR signaling [21]. Furthermore, SARS‐CoV‐2 stimulation of monocyte‐derived cells (DCs and macrophages) from healthy donors did not cause induction of IFN production (type I, II, and III), potentially due to viral antagonism of STAT1 phosphorylation [75]. Neither pDCs nor monocyte‐derived DCs appear to be productively infected by SARS‐CoV‐2 [75, 76]. Host genetics are likely important, as studies have shown inborn errors of type I IFN signaling, autoantibodies against type I IFNs, and X‐linked TLR‐7 deficiency in a subset of severe COVID‐19 cases [77, 78, 79].

## M‐MDSCs are expanded in circulation during acute COVID‐19

The emergency myelopoiesis described in patients with COVID‐19 [22, 25, 64] indicates that expansion of M‐MDSCs may occur in the bone marrow. M‐MDSCs can be identified as CD14+HLA‐DR^–/lo^ cells [80, 81, 82], and may therefore not always be distinguished from CMs. Several cytokines could be involved in the expansion of M‐MDSCs during COVID‐19, including IL‐6 and IL‐10 [83]. Plasma IL‐6 has been reported to increase with increased disease severity in patients with COVID‐19 [23, 26, 84], and increased IL‐6 levels associate with decreased HLA‐DR expression on CD14+ monocytes [84]. Elevated levels of GM‐CSF could also contribute to M‐MDSC development [20, 85]. Severe COVID‐19 likely induces both downregulation of HLA‐DR on monocytes [86] and expansion of M‐MDSC in the bone marrow [26, 68].

Multiple studies have found an expansion of M‐MDSCs/MDSC‐like cells in patients with severe COVID‐19, characterized by low expression of HLA‐DR and CD86 and increased ROS production and S100A8/A9 [4, 24, 26, 27, 42, 64, 82, 87]. M‐MDSCs expand early during severe COVID‐19, are elevated at hospitalization (49), and normalize during convalescence [24, 26]. Moreover, we showed that M‐MDSC levels within 2 weeks since symptom onset predicted subsequent disease severity [26].

Due to the lack of surface markers unique to M‐MDSCs, functional analysis of T‐cell suppression is important to confirm phenotypic identification [13]. We showed that M‐MDSCs isolated from patients with COVID‐19 suppress CD4 and CD8 T‐cell proliferation and IFN‐γ secretion in vitro, and that supplemental L‐arginine decreases this suppressive effect [26]. Furthermore, Arg‐1 levels were increased both in M‐MDSC cocultures and in plasma from patients with COVID‐19 [26]. In line with this, Reizine et al. demonstrated increased Arg‐1 activity and decreased arginine levels in plasma during severe COVID‐19 and a negative correlation between M‐MDSC levels and T‐cell counts [82]. The expansion of M‐MDSCs could be an important factor in the T‐cell alterations during severe COVID‐19, characterized by decreased levels, exhaustion, and changed metabolic profiles [1, 26, 67, 87].

In summary, monocytes and DCs in blood display altered frequencies in COVID‐19 with a pronounced reduction of DC numbers and a decreased capacity to respond functionally to stimulation, and this seems to be associated with disease severity. In addition, levels of blood M‐MDSCs are expanded, further contributing to the blunted immune response described in many studies.

## Early immune responses in the upper respiratory tract appear to be essential for subsequent disease severity

SARS‐CoV‐2 enters the upper airways through respiratory droplets [88], and an early robust innate immune response in the nasopharynx is likely crucial to limiting the infection. So far, few studies have investigated monocytes, DCs, and M‐MDSCs in the upper airways in COVID‐19, despite this being the primary site of infection [89].

In general, patients with moderate to severe COVID‐19 display low type I IFNs in blood, and a weak/delayed IFN induction upon SARS‐CoV‐2 infection is also supported by virological studies [90, 91]. Galani et al. showed that in moderate to severe COVID‐19 patients, type I and III IFN responses were delayed and reduced compared to influenza patients, and instead, pro‐inflammatory cytokines such as IL‐6 and IL‐8 were produced first during COVID‐19, in contrast to the conventional kinetics of viral infections. A later induction of IFNs occurred in a subset of patients, after progression to severe disease [92]. Similarly, Hadjadj et al. found that low IFN‐α and undetectable IFN‐β in blood was associated with blood viral load, increased levels of IL‐6 and TNF, and severe disease [93]. However, these studies focused on blood, and not the site of infection.

Using nasopharyngeal swabs and single‐cell RNA sequencing (scRNA‐seq), Ziegler et al. found that epithelial cells from patients with mild/moderate COVID‐19 expressed an IFN‐responsive gene signature compared to severe disease [94]. It is unclear whether the type I IFN response is affected in patients with mild COVID‐19. Patients with asymptomatic SARS‐CoV‐2 infection associated with higher levels of plasma IFN‐α compared to hospitalized patients [69]. In contrast, Vu et al. did not find elevated nasopharyngeal levels of IFN‐α in patients with relatively mild COVID‐19, but instead an increase in CCL2 and IL‐8 within the first week since symptom onset [95]. Similarly, IFN‐α was not increased in NPA samples from patients with COVID‐19, in contrast to patients with influenza A and B and RSV, indicating that the lack of IFN‐α is SARS‐CoV‐2 specific [12].

Early events at the site of SARS‐CoV‐2 infection likely influence the subsequent disease course (Fig. 2 and Table 1), and more studies are needed to understand the kinetics of the immune response in the upper respiratory tract, what cell types are involved, and how this relates to disease severity.

## Hyperinflammation and monocyte infiltration in the lower respiratory tract during severe COVID‐19

Severe COVID‐19 is characterized by hypoxic respiratory failure due to a ventilation‐perfusion mismatch and an increased diffusion barrier caused by alveolar inflammation (Fig. 2) [96]. Histopathological analyses of lung autopsy samples have shown diffuse alveolar damage and fibroproliferative remodeling [97]. Furthermore, studies have found elevated levels of pro‐inflammatory cytokines and chemokines in the lungs compared to blood during severe COVID‐19 [32, 42], supporting a hyperinflammatory milieu in the lungs. The dominant immune cell types in the lungs of patients with severe or fatal COVID‐19 are myeloid cells—including monocytes, macrophages, and neutrophils [32, 33, 97, 98, 99]—and the enrichment of monocytes seems to be—to some extent—COVID‐19 specific [98, 100].

Monocytes are likely recruited from the blood to the lower airways during COVID‐19 [28, 32, 33]. Szabo et al. found a phenotypic overlap between monocytes in blood and airways with decreased frequencies of conventional CD163^hi^ macrophages [32]. Transcriptional analysis also showed a dominance of infiltrating monocytes transitioning into macrophages during the first four weeks of acute respiratory distress syndrome, and a later repopulation of alveolar macrophages [97]. Similarly, single‐nucleus RNA‐seq on lung tissue samples from fatal COVID‐19 cases revealed increased levels of alveolar macrophages, monocyte‐derived macrophages and monocytes with expression of several genes indicative of aberrant activation compared to controls [101].

Elevated levels of CCL2 and CCL7 in BAL fluid from patients with severe COVID‐19 [23, 32, 33] provide a mechanism of monocyte recruitment. CCL2 is mainly produced by monocytes and macrophages and is an important chemokine for monocyte migration and infiltration [102]. Increased plasma CCL2 levels also associate with COVID‐19 mortality [103], and patients with severe COVID‐19 display elevated expression of CCR2 on blood monocytes and cDCs [68].

Monocytes recruited to the lower airways likely contribute to the immunopathology [28, 32, 33], and scRNA‐seq of BAL cells has revealed elevated frequencies of proinflammatory monocyte‐derived macrophages in patients with severe COVID‐19 compared to patients with moderate disease [33]. BAL monocytes/macrophages also showed enrichment in several immune pathways—including response to cytokines, IFNs, hypoxia, and Fc‐receptor signaling—and expressed high transcript levels of cytokines and chemokines including IL‐6, IL‐1β, IL‐18, IL‐10, and CCL2 compared to blood cells [42]. Similarly, Szabo et al. showed increased chemokine transcripts in respiratory myeloid cells as opposed to blood [32]. In contrast, important pathways including clearance of apoptotic cells, lipid metabolism, and antigen presentation were downregulated in respiratory myeloid cells [42]. These monocyte‐derived macrophages also display a profibrotic phenotype, closely associated with a profibrotic milieu in the lungs during severe COVID‐19 and show transcriptomic similarities with macrophages from idiopathic pulmonary fibrosis patients [97].

The duration of inflammation and the potential association with long‐term COVID‐19 symptoms remains unknown. The deficiency in pDC numbers and function seems to persist after 7 months in hospitalized patients [74]. In a recent study, COVID‐19 patients with respiratory symptoms 3–6 months after discharge had increased concentrations of proteins related to apoptosis, tissue repair, and epithelial injury. Furthermore, BAL cells were increased and had an altered composition compared to controls, with elevated numbers of alveolar macrophages, T cells, and B cells. Several cell types were associated with reduced pulmonary function and radiographic abnormality, including IMs and NCMs. Levels of IMs and NCMs seem to decrease after 1 year [104].

Less is known about DCs in the lower airways during COVID‐19, and decreased numbers in blood could indicate recruitment to the site of infection. Perez‐Gomez et al. observed that a number of inflammatory markers were inversely correlated with the percentage of blood DCs expressing integrin β7, and speculated that DC migration might be important in hyperinflammation [74]. CCR2 ligands including CCL2 could mediate recruitment of pre‐cDCs to the airways, as recently demonstrated in mice with influenza [105]. However, recruitment of DCs is not established. Sánchez‐Cerillo et al. found that IMs, NCMs, and cDC2s were enriched in bronchial aspirates compared to matched blood samples, whereas pDCs or cDC1s were not found [28]. However, control samples for comparison would be desirable. Comparing moderate and severe COVID‐19 patients, scRNA‐seq displayed lower frequencies of cDC and pDC in severe patients [33]. The extent and implications of DC recruitment to the lungs warrants further investigation.

## SARS‐CoV‐2 does not seem to cause productive infection in macrophages

SARS‐CoV‐2 infects airway epithelial cells by binding to the cell surface receptor ACE2 [106]. It has been suggested that SARS‐CoV‐2 can cause abortive infection of monocyte‐derived cells (macrophages and DCs), inducing type I IFNs, proinflammatory cytokines, and cell death [107, 108]. Another study, based on scRNA‐seq, suggested that alveolar macrophages may support viral replication and contribute to the spread of the virus [98]. However, direct evidence of productive infection of macrophages is lacking. Interestingly, a recent preprint shows that human monocyte‐derived macrophages and tissue‐resident alveolar macrophages do not express sufficient levels of ACE2 to enable viral entry [109]. ACE2 overexpression in macrophages caused entry of SARS‐CoV‐2 and early stage replication, but not release of virions, which was only achieved after blocking of IFN signaling. The absence of a pro‐inflammatory and IFN response from macrophages after exposure to SARS‐CoV‐2 in vitro may indicate impaired viral sensing, which could enable replication for a longer time in epithelial cells before cell‐extrinsic danger signals are sensed by macrophages. This could cause an extensive, but delayed, pro‐inflammatory response [109].

Interestingly, recently published data demonstrate that Fcγ receptor (mainly CD16) mediates uptake of SARS‐CoV‐2, causing abortive infection in monocytes and macrophages. Infection leads to activation of the NLRP3 inflammasome and pyroptosis, resulting in the release of pro‐inflammatory cytokines [110, 111]. Importantly, this could be a mechanism behind the severe inflammation seen in the lungs of patients with severe COVID‐19.

## Respiratory M‐MDSCs

Studies on respiratory M‐MDSCs have been scarce. scRNA‐seq of BAL cells from patients with severe COVID‐19 demonstrated downregulation of HLA‐DRA and HLA‐DRB1 in monocytes/macrophages [25]. Szabo et al. found elevated frequencies of HLA‐DR^lo^ CMs in blood compared to HCs, and lower frequencies of a corresponding cell type in airways from patients with severe COVID‐19 [32]. Kvedaraite et al. found evidence of M‐MDSCs at the site of infection from a publicly available BAL scRNA‐seq dataset [27, 33]. We did not, however, find increased frequencies of M‐MDSCs in NPA or ETA from COVID‐19 patients compared to paired blood samples using flow cytometry [26], but the lack of control ETA samples makes interpretation more difficult. In contrast, influenza patients had elevated frequencies of M‐MDSCs in NPA compared to healthy individuals [26]. Collectively, these data indicate that M‐MDSCs are more frequent in blood than in the airways in COVID‐19 patients, which is in line with observations of a hyperinflammatory milieu in the lungs. However, it is possible that M‐MDSCs are recruited to other parts of the airways, and/or that M‐MDSCs differentiate to macrophage‐like cells, as seen after migration to tumor sites [112]. More studies are needed to understand the role of M‐MDSCs in the airways during COVID‐19.

In summary, evidence suggests an insufficient initial response to SARS‐CoV‐2 in airways, potentially contributing to more severe disease and the recruitment of monocytes to the lungs of patients with severe COVID‐19, where they can differentiate into monocyte‐derived macrophages and contribute to inflammation (Fig. 2 and Table 1). The hyperinflammatory status of these cells supports the notion that monocytes/macrophages are more activated in the airways compared to the blood. However, since functional analyses of respiratory monocytes and macrophages in COVID‐19 are scarce, most findings are based on RNA‐seq.

## Induction of adaptive immune responses during COVID‐19

In order to clear respiratory viral infections, adaptive immune responses are typically essential. In SARS‐CoV‐2 infection, it has been suggested that early induction of virus‐specific T cells is associated with control of viral replication and milder disease, while delayed T‐cell activation associates with more severe cases [113]. T‐cell frequencies in the airways are also altered in patients with severe COVID‐19 [32, 100], and Szabo et al. found an association between higher airway T‐cell frequency and younger age and survival in COVID‐19 [32]. This finding is further supported by the absence of increased T‐cell frequencies in the lungs of patients with fatal COVID‐19 [101].

The induction of antigen‐specific T cells is dependent on antigen presentation. Both cDCs and monocytes are recruited to the airways during respiratory infections and contribute to antigen trafficking to lymph nodes, where they participate in antigen presentation to T cells, though the relative contribution of monocytes is unclear [18, 114, 115]. In contrast, MDSCs suppress T cells, hampering the initiation of adaptive immune responses [20]. Many of the observed changes—including decreased levels of circulating cDCs, low expression of costimulatory markers and HLA‐DR, as well as decreased functional capacity in combination with elevated levels of MDSCs in blood from COVID‐19 patients—appear more pronounced in severe disease, which could indicate a severity‐associated decrease in antigen presentation. Less is known about the respiratory tract, but reduced frequencies of cDCs in BAL may indicate that tissue homing and potentially antigen presentation could be affected in cDCs from COVID‐19 patients [33]. However, cDCs could also be located in other compartments of the airways (not the luminal space) or in lymph nodes.

To what extent DCs are recruited to the airways during severe COVID‐19, subsequently migrate to lymph nodes, and ultimately activate T cells remains to be investigated.

## Concluding remarks

Our knowledge of the immune response to SARS‐CoV‐2 infection has increased rapidly, and despite the challenges in comparing results across studies, a picture of the immune response has emerged. A lack of early IFN responses and a more pro‐inflammatory response at the site of infection, low levels of pDCs, and presence of IFN auto‐antibodies are likely important in patients that go on to develop severe disease. These early events may lead to an inability to control viral replication and increased inflammation in the respiratory tract, along with a blunted response in blood immune cells. Monocytes are recruited to the lower airways of patients with severe COVID‐19, causing excess inflammation. Decreased levels of cDCs and increased levels of M‐MDSCs in blood may contribute to delayed T‐cell responses in severe cases of COVID‐19.

The majority of studies focus on the phenotype and function of circulating monocytes, DCs, and M‐MDSCs, but less is known about their respiratory counterparts. Not surprisingly, functional studies on COVID‐19 respiratory cells are almost entirely lacking, which is unfortunate as they could shed light on the local immune activation, likely an important driver of disease progression. M‐MDSCs appear to be important predictors of disease severity, but considerable challenges remain in defining these cells. Furthermore, due to the difficulty of obtaining samples early in the disease course, many studies focus on later events. Longitudinal studies could shed more light on the immune response over time, including IMs in mild versus severe disease. Moreover, the role of monocytes, DCs, and M‐MDSCs in the progression of long COVID is still largely unknown.

The rapid evolution of the study of SARS‐CoV‐2 has thrown into sharp relief the gaps in our knowledge of the pathogenesis of other respiratory viral infections—in particular, the immune response at the site of infection—and untangling which aspects of COVID‐19 pathogenesis are unique compared to other respiratory infections is likely to take many years.

## Conflict of interest

Sara Falck‐Jones and Björn Österberg declare no conflict of interest. Anna Smed‐Sörensen is a consultant for AstraZeneca.
